# Modification of non-conservative double-strand break (DSB) rejoining activity after the induction of cisplatin resistance in human tumour cells

**DOI:** 10.1038/sj.bjc.6690135

**Published:** 1999-02

**Authors:** R A Britten, S Kuny, S Perdue

**Affiliations:** Department of Oncology, University of Alberta, Cross Cancer Institute, 11560 University Avenue, Edmonton, Alberta, Canada

**Keywords:** γ-radiation, cisplatin resistance, DNA repair, repair fidelity, human ovarian tumour cells

## Abstract

The induction of collateral radioresistance after the development of cisplatin resistance is a well-documented phenomenon; however, the exact processes that are responsible for the cisplatin-induced radioresistance remain to be elucidated. There was no obvious difference in the level of radiation-induced DNA double strand breaks (DSBs), in DSB rejoining rates, or the level of the catalytic subunit of the DNA-dependent protein kinase (DNA-PKcs) in the cisplatin- and radiation-sensitive 2780/WT and cisplatin-resistant 2780/CP cell lines. However, there was a significantly (*P*< 0.01) lower level of DSB misrejoining activity within nuclear protein extracts derived from the cisplatin-and radiation-sensitive 2780/WT and OAW42/WT tumour cell lines than in similar extracts from their cisplatin- (and radiation-) resistant 2780/CP and OAW42/CP counterparts. All of the DSB misrejoining events involved deletions of between 134 and 444 bp that arose through illegitimate recombination at short repetitive sequences, such as those that arise through non-homologous repair (NHR). These data further support the notion that the radiosensitivity of DSB repair proficient human tumour cell lines may be partly determined by the predisposition of these cell lines to activate non-conservative DSB rejoining pathways. Furthermore, our data suggest that the induction of acquired cisplatin resistance is associated with a two- to threefold decrease in the activity of a non-conservative DSB rejoining mechanism that appears to be a manifestation of NHR. © 1999 Cancer Research Campaign


*British Journal of Cancer* (1999) **79**(5/6), 843–849
© 1999 Cancer Research Campaign
Article no. bjoc.1998.0135

### Modification of non-conservative double-strand break (DSB) rejoining activity after the induction of cisplatin resistance in human tumour cells

**RA Britten, S Kuny and S Perdue**

Department of Oncology, University of Alberta, Cross Cancer Institute, 11560 University Avenue, Edmonton, Alberta, Canada

**Summary** The induction of collateral radioresistance after the development of cisplatin resistance is a well-documented phenomenon; however, the exact processes that are responsible for the cisplatin-induced radioresistance remain to be elucidated. There was no obvious difference in the level of radiation-induced DNA double strand breaks (DSBs), in DSB rejoining rates, or the level of the catalytic subunit of the DNA-dependent protein kinase (DNA-PKcs) in the cisplatin- and radiation-sensitive 2780/WT and cisplatin-resistant 2780/CP cell lines. However, there was a significantly ($P < 0.01$) lower level of DSB misrejoining activity within nuclear protein extracts derived from the cisplatin- and radiation-sensitive 2780/WT and OAW42/WT tumour cell lines than in similar extracts from their cisplatin- (and radiation-) resistant 2780/CP and OAW42/CP counterparts. All of the DSB misrejoining events involved deletions of between 134 and 444 bp that arose through illegitimate recombination at short repetitive sequences, such as those that arise through non-homologous repair (NHR). These data further support the notion that the radiosensitivity of DSB repair proficient human tumour cell lines may be partly determined by the predisposition of these cell lines to activate non-conservative DSB rejoining pathways. Furthermore, our data suggest that the induction of acquired cisplatin resistance is associated with a two- to threefold decrease in the activity of a non-conservative DSB rejoining mechanism that appears to be a manifestation of NHR.

**Keywords:** γ-radiation; cisplatin resistance; DNA repair; repair fidelity; human ovarian tumour cells

The inherent cellular capacity to repair radiation-induced DNA damage is one of the most important determinants of radioresistance. Although DNA double-strand break (DSB) repair pathways are unquestionably important in determining the lethality of radiation-induced DNA damage (Giaccia et al, 1985; Taccioli et al, 1994; Kirchgessner et al, 1995; Lees-Miller et al, 1995), DSB rejoining capacity does not correlate well with the relative radioresistance of human tumour cell lines (reviewed in Olive et al, 1994). This disparity between DSB rejoining capacity and cellular radiosensitivity in human tumour cells may be partly attributable to the inability of the currently available techniques to dissociate complex DSBs (that are likely to have the most profound biological consequences) from less innocuous DSBs. An alternative explanation for this disparity is that the majority of the techniques used to measure DSB induction and rejoining give no information on the fidelity of DSB rejoining. Thus, cells that have a high DSB rejoining capacity may not necessarily be repairing DSBs efficiently, and could even be generating chromosomal aberrations during DSB rejoining. Interestingly, in contrast to DSB rejoining levels, the level of radiation-induced chromosomal aberrations correlates well with radiosensitivity in both tumour (Lambin et al, 1994; Sasai et al, 1994) and fibroblast (Russell et al, 1995) cells.

*Received 8 June 1998*
*Accepted 24 July 1998*

*Correspondence to:* RA Britten, Division of Experimental Oncology, Department of Oncology, Cross Cancer Institute, 11560 University Avenue, Edmonton, Alberta T6G 1Z2, Canada

We have recently reported that DSB rejoining fidelity significantly ($P < 0.001$) correlates with the clinically relevant radiosensitivity, i.e. $SF_2$, of eight human tumour cell lines (Britten et al, 1997). Nuclear protein extracts from radiosensitive, yet overtly DSB rejoining proficient, tumour cells were found to be less capable of correctly rejoining *Eco*RI-induced DSBs than were similar extracts from radioresistant tumour cells. The fidelity with which restriction endonuclease-induced DSBs are repaired within the intact cellular environment also varies with respect to $SF_2$ values in human tumour cell lines, with the most radioresistant cell lines exhibiting a higher fidelity of DSB repair than their radiosensitive counterparts (Powell and McMillan, 1991; Powell et al, 1992, 1994). It has been suggested that restriction endonuclease-generated DSBs act as substrates for proteins that are involved in the (abnormal) conversion of DSBs into chromosomal aberrations (Bryant and Liu, 1994). This conclusion is supported by the fact that ataxia telangiectasia (AT) and *irs* cells exhibit high DSB misrejoining activity (Cox et al, 1984; Thacker and Ganesh, 1990; Ganesh et al, 1993; Powell et al, 1993; Lou et al, 1996), and generate high levels of chromatid-type aberrations in response to restriction endonuclease-induced DSBs (Liu and Bryant, 1993; Bryant, and Liu, 1994).

The biochemical basis for the differential level of DSB misrejoining activity in nuclear protein extracts from radiosensitive and radioresistant tumour cells is presently unknown, although we have shown that the high DSB misrejoining activity in radiosensitive tumour cells was not a consequence of non-specific endonuclease activity but rather due to an increase in non-conservative DSB rejoining activity that results in losses of between 40 and 440 bp of DNA (Britten et al, 1997). Furthermore, previous studies have shown that the majority of DSB misrejoining events generated by AT cellular protein extracts were primarily deletions arising from illegitimate recombination, i.e. the DNA recombines at short sequence repeats that are frequently far apart (Thacker et al, 1992).

These data raise the possibility that the radiosensitivity of DSB rejoining-proficient human tumour cell lines may be partly determined by their predisposition to activate non-conservative DSB rejoining pathways. Conversely, a radioresistant phenotype might arise through the suppression (either inherent or environmentally induced) of such rejoining pathways. The testing of such a hypothesis requires a model system consisting of genetically matched cell lines and the ability to stably induce a radioresistant phenotype. We, and others, have reported that the induction of cisplatin resistance frequently (but not invariably) leads to the induction of collateral radioresistance in human tumour cell lines (Louie et al, 1985; Hida et al, 1989; Twentyman et al, 1991; Britten et al, 1993, 1994). Interestingly, the induction of collateral radioresistance only occurs in those cisplatin-resistant cells that were derived from inherently radiosensitive parental cells (Britten et al, 1994). Furthermore, the level of cisplatin-induced radioresistance was independent of the level of cisplatin resistance induced, but highly dependent upon the inherent radiosensitivity of the parental cell lines with the most radiosensitive cells showing the greatest degree of cisplatin-induced collateral radioresistance (Britten et al, 1994). The possibility, therefore, exists that the induction of a radioresistant phenotype after the development of cisplatin resistance in radiosensitive tumour cell lines is a consequence of cisplatin-induced changes in the DNA damage-response pathways, which manifest themselves through alterations in the fidelity of DSB rejoining. We, thus, determined whether the induction of collateral radioresistance in the human 2780 and OAW42 ovarian cancer cell lines was associated with an alteration in the level and/or fidelity of DSB rejoining.

### MATERIALS AND METHODS

### Cell lines

The 2780/WT and OAW42/WT cell lines are single-cell derived cell lines that we established from the human A2780 and OAW42 ovarian tumour cell lines respectively. The cisplatin-resistant variants 2780/CP and OAW42/CP were developed from the 2780/WT and OAW42/WT cell lines, respectively, by stepwise incubation with cisplatin. Relative to their respective parental cell lines, the 2780/CP and OAW42/CP cell lines are three- and 3.7-fold more resistant to clinically relevant (i.e. 1 $\mu$g ml$^{-1}$) cisplatin concentrations (Table 1). Both the parental and cisplatin-resistant variants were routinely maintained as log-phase monolayer culture in Dulbecco's modified Eagle medium (DMEM)/F12 medium (Life Technologies, Grand Island, NY, USA) supplemented with 10% fetal calf serum (FCS: Life Technologies) and 1% penicillin–streptomycin at 37°C in a humidified 5% carbon dioxide/95% air atmosphere.

Chinese hamster ovary (CHO) cells were used as a reference cell line to facilitate interlaboratory comparisons. The level of DSB misrejoining activity in CHO (strain AA8) cells has been previously characterized (Britten et al, 1997), and we have also established their susceptibility to DSB induction after exposure to $^{60}$Co $\gamma$-rays (Murray et al, 1994). The CHO-AA8 cells were maintained in monolayer culture in McCoy's 5A medium (Hsu's modification; Life Technologies) supplemented with 10% FCS and antibiotics and passaged every 2–3 days to ensure exponential growth.

**Table 1** Characterization of the radiation and cisplatin sensitivity of the human ovarian tumour cell lines and CHO-AA8 cells

| | γ-Rays | | | | Cisplatin |
|---|---|---|---|---|---|
| **Cell line** | **$\alpha$ (Gy$^{-1}$)** | **$\beta$ (Gy$^{-2}$)** | **$SF_2$** | **$D_{0.1}$ (Gy)** | **$SF_1$[a]** |
| 2780/WT | 0.363 (0.043)[b] | 0.056 (0.006) | 0.38 | 3.95 | 0.29 |
| 2780/CP | 0.343 (0.038) | 0.011 (0.009) | 0.48 | 5.68 | 0.88 |
| OAW42/WT | 0.549 (0.042) | 0.015 (0.005) | 0.31 | 3.79 | 0.22 |
| OAW42/CP | 0.261 (0.006) | 0.025 (0.006) | 0.53 | 5.73 | 0.81 |
| CHO-AA8 | 0.154 (0.022) | 0.032 (0.002) | 0.64 | 6.41 | 0.91 |

[a]Fraction of cells surviving exposure to 1 $\mu$g ml$^{-1}$ cisplatin. [b]Standard error of mean.

### Radiation clonogenic cell survival assays

The radiosensitivity of the cell lines used in this study have been previously established (Britten et al, 1993, 1994). However, to ensure that the radiosensitivity of the cells recovered from frozen storage had not altered and to facilitate a better statistical comparison between radiosensitivity and DNA repair fidelity, complete radiation survival curves (over the range 0–10 Gy) were constructed on the same batch of cell lines used to prepare the nuclear protein extracts. To ensure that the radiation sensitivity determined on this occasion was not erroneous, radiation sensitivity was determined a further two times in the subsequent 7 days. The radiosensitivity parameters presented in Table 1 represent the pooled data from all three survival curve experiments. In no instance did we find that the radiation sensitivity determined on the day that the protein extract was prepared varied from either historical data, or from that observed in the subsequent assays. The procedure followed for these experiments is outlined below. On the day of the experiment, the cell monolayers were washed with sterile prewarmed (37°C) PBS, and detached using 0.25% trypsin/1 mM EDTA at 37°C. The cell pellets were then washed twice with warm PBS. Two-thirds of the cell pellet was used to prepare the nuclear protein extract (see below) whereas the other third was used to determine radiosensitivity: the cells were resuspended in DMEM/F12 media (supplemented with 10% FCS and antibiotics), and seeded at densities of between $10^2$ and $5 \times 10^4$ cells per 60 mm tissue culture dish. Triplicate plates were set up for each of the five radiation dose levels, placed in aluminium irradiation chambers and irradiated using a $^{60}$Co irradiator at a dose rate of 6 Gy min$^{-1}$. After 15 days at 37°C (5% carbon dioxide/95% air), the colonies were fixed in 70% ethanol and stained with 10% methylene blue, and those colonies containing greater than 50 cells were counted.

### Data handling and presentation

The experimental data were fitted to the linear-quadratic equation:

$$-\mathrm{Ln}\ S = \alpha D + \beta D^2$$

In which $S$ is equivalent to the surviving fraction at a given dose, D, and $\alpha$ and $\beta$ are constants. The data were fitted to a linear-quadratic function using the non-linear regression program of the Prizm software package (Graphpad Software, San Diego, CA, USA).

 *© Cancer Research Campaign 1999*

### Measurement of γ-ray-induced DNA double-strand breaks

A slightly modified version of our previously published pulsed field gel electrophoresis (PFGE) technique (Murray et al, 1994) was used to determine the level of γ-ray-induced DSBs. The initial level of DSBs induced over the dose range 0–10 Gy was determined by immediately embedding the irradiated tumour cells in InCert agarose (FMC, Rockland, ME, USA; 1% solution in PBS), and then incubating the agarose plugs in 5 ml of ESP (500 mM EDTA, 1% w/v sarkosyl, 1 mg $ml^{-1}$ proteinase K; pH 9) for 24 h at 47°C. The DSB repair capacity of the cell lines was determined by measuring the disappearance of DSB over a 240-min time period. Agarose plugs were prepared as described above using tumour cells that had either just been irradiated with 30 Gy or that had been left at 37°C for 10, 30 or 240 min. After three washes (for 1 h each) in sterile 0.5 × TBE buffer (45 mM Tris base, 45 mM boric acid, 1 mM EDTA; pH 7.2), the DNA in the plugs (~5 μg from ~9 × $10^4$ cells) was separated using a CHEF-DR II system (Bio-Rad, Mississauga, Ontario, Canada). The DNA was separated for 18 h by a 40-V (~1.21 V $cm^{-1}$) electric field, with a switch time of 75 min and a field angle of 120°. The buffer (0.5 × TBE) was recirculated through a temperature-regulating unit maintained at 25°C.

After electrophoretic separation, the DNA was transferred to Hybond-N+ nylon membranes (Amersham, Arlington Heights, IL, USA) by capillary blotting, and baked at 80°C for 2 h. After prehybridization of the membranes for 4 h at 45°C in Hybrisol I (Oncor, Gaithersburg, MD, USA), the membrane was hybridized with $^{32}$P-labelled human Alu+ probe for 18 h at 42°C. Unbound probe was removed by washing membranes three times in 2 × SSC/0.1% SDS at room temperature and twice in 0.2 × SSC/0.1% SDS at 20°C. The distribution of radioactivity between the plug and lane was then determined using a GS-250 Molecular Imager (Bio-Rad).

### DNA repair fidelity assay

The fidelity of DSB repair effected by nuclear protein extracts from the cell lines was assessed using a slightly modified version of our cell-free plasmid reactivation assay (Britten et al, 1997). Briefly, this plasmid reactivation assay determines the ability of nuclear protein extracts to correctly rejoin a simple staggered (*Eco*RI-induced) DSB within the *lac*Zα complementation gene of the pUC18 plasmid. DNA repair fidelity is scored by the loss of *lac*Zα gene function (α-complementation of β-galactosidase): misrejoining of the DSBs will result in a mutated *lac*Zα sequence that will produce colourless colonies when transfected into *Escherichia coli*, whereas the correct rejoining will lead to the production of blue colonies. An uncut ampicillin-resistance gene (*amp*$^1$) acts as a selectable marker for transfection efficiency, and an internal control for non-specific nuclease activity.

Nuclear protein extracts were prepared from the cell lines as previously described (Britten et al, 1997). The experimental conditions used to derive the data presented in this paper are described below. Two micrograms of linearized plasmid DNA and 1 μg of nuclear protein extract were mixed in 50 μl containing 50 mM tris-HCl (pH 7.6), 10 mM magnesium chloride, 1 mM ATP, 1 mM DTT, 50 μM dNTPs and 5% (w/v) polyethylene glycol-8000, and incubated at 18°C for 24 h. The plasmid DNA was then isolated by spinning down the reaction mixture through Ultrafree-Probind Filter column (Millipore Corporation, Bedford, MA, USA). For quality control purposes, an aliquot was removed and treated with T4-ligase (Life Technologies) before being further processed.

The recovered plasmid was diluted fivefold with TE buffer (pH 7.6) and then transfected into the *E. coli* DH5α strain using the Gibco-BRL protocol for DH5α bacteria. The transformed cells were then plated out on premade LB plates [LB medium/0.7% agar containing 100 μg $ml^{-1}$ ampicillin, 20 mg $ml^{-1}$ X-gal (5-bromo-4-chloro-3-indolyl-β-D-galactopyranoside) and 200 mg $ml^{-1}$ of IPTG (an inducer of *lac*Zα gene function)]. An aliquot of cells was also plated out on LB plates that contained no ampicillin to determine bacterial cell viability/plating efficiency. After overnight incubation at 37°C, and a subsequent 4-h incubation at 4°C the number of blue and white colonies (that were greater than 1 mm in diameter) was determined. To ensure that our results were not attributable to experimental artefacts, it was necessary to undertake several quality control experiments. To guard against the possibility of misidentifying 'white' colonies, it was necessary to restreak the 'mutant' colonies onto fresh selection medium to verify the mutation in the *lac*Zα gene. It was also necessary to determine bacterial cell viability (in the absence of ampicillin) to establish whether nuclease activity was affecting the integrity of the *amp*$^r$-gene, and to correct for variations in bacterial cell plating numbers. Moreover, to correct for the presence of small amounts of undigested plasmid, ligation activity within the bacteria (although as linearized plasmid molecules do not transform bacteria very well this was not a major concern) and importantly any DNA modification by the bacteria, we performed transformation experiments using *Eco*RI-digested plasmid that had not been exposed to nuclear protein extracts. Before analysis, the number of 'spontaneously' arising colonies were subtracted from the number of colonies arising from the extract-treated plasmid.

Comparative studies on the fidelity of DSB rejoining effected by nuclear protein extracts from the radiosensitive and radioresistant cell lines were performed simultaneously using the same batch of competent cells, reagents and *Eco*RI-digested plasmid DNA. A minimum of three experiments were performed with each nuclear protein extract, the results presented in this paper represent the pooled data from these experiments.

### DNA sequencing

Plasmid DNA was isolated from six randomly selected white and five blue clones generated after exposure to the nuclear protein extract of the 2780/WT cell line. The region of the pUC18 plasmid corresponding to nt. 150–777 [which we have previously shown to encompass the majority of deletional events (Britten et al, 1997)] was then polymerase chain reaction (PCR) amplified using a GeneAmp PCR system 9600 (Perkin Elmer) and the following set of primers: forward, 5′-CGGCA TCAGA GCAGA TTGTA-3′ (primer 2F); reverse, 5′-TGGAT AACCG TATTA CCGCC-3′ (primer 2R); and the following PCR conditions: 95°C, 2 min; 40 cycles of 95°C, 15 s, 60°C, 30 s, 72°C, 15 s; 72°C, 7 min. We subsequently sequenced the DNA from the white clones to ascertain the nature of the mutations in the *lac*Zα gene. Primers 2 F and 2R were used to sequence the transcribing and non-transcribing strand, respectively, using the Prism ABI Dye terminator sequencing kit (Perkin Elmer) in accordance with the manufacturer's instructions with the following PCR conditions: 25 cycles of 96°C, 10 s, 50°C, 5 s, 60°C, 4 min in a GeneAmp PCR system 9600.

### Western blotting

A recent report suggests that cisplatin–DNA adducts (especially DNA interstrand cross-links) inhibit the activity of DNA-PK

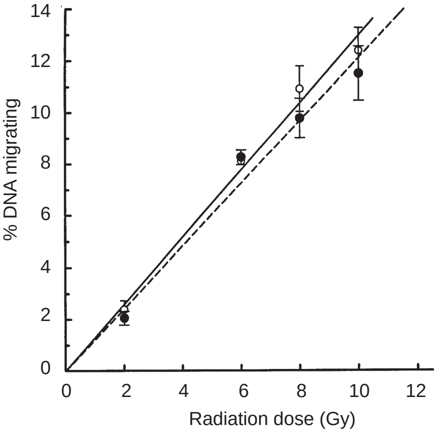

**Figure 1** PFGE-induced elution of DNA (% DNA migrating) from plugs containing embedded nuclei of 2780/WT (○) and 2780/CP (●) cells that had been exposed to graded doses of $^{60}$Co γ-radiation. DNA elution at each dose point was assayed by at least three separate experiments, each experiment consisting of at least four replicate dishes

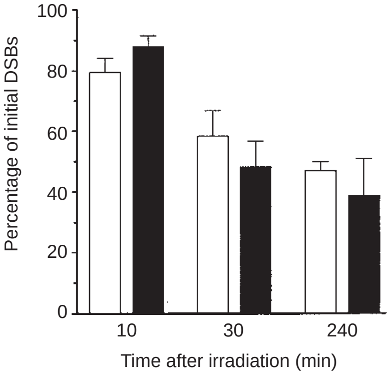

**Figure 2** The rejoining of radiation (30 Gy of $^{60}$Co γ-rays)-induced DNA DSB with respect to time elapsed from irradiation in 2780/WT (open bar) and 2780/CP (solid bar) cells

(Turchi et al, 1997). These data suggest that the modification of radioresistance after the development of cisplatin resistance may arise through the constitutive up-regulation of DNA-PK activity. We, thus, determined the level of the catalytic subunit of the DNA-dependent protein kinase (DNA-PKcs) in nuclear protein extracts from 2780/WT and 2780/CP cells. The protein content of the 2780/WT and 2780/CP nuclear protein extracts was adjusted to 3 μg $\mu l^{-1}$ and then denatured by boiling the protein in the presence of 1.5% sodium dodecyl sulphate (SDS) for 5 min, 40 μg of each nuclear protein extract was then electrophoretically (200 V) separated on a 7% polyacrylamide gel maintained at 20°C. The separated protein bands were transferred to nitrocellulose membranes using a Biorad Mini Transblot apparatus (Bio-Rad) for 1.5 h at 75 V (maintained at 20°C). The membranes were then incubated for 4 h in blocking buffer [PBS, 0.1% v/v Tween-20, 5% (w/v) skim-milk powder], and then incubated in the presence of a (1:3000) rabbit anti-DNA-PKcs antibody (kindly supplied by Dr Allalunis-Turner) overnight at 4°C with gentle agitation. The blots were then washed four times (10 min each) in TBST (20 mM Tris, pH 7.5, 150 mM sodium chloride, 0.05% Tween-20) and incubated with a 1:2500 dilution of HRP-tagged goat anti-rabbit antibody (Santa Cruz Biotechnology, Santa Cruz, CA, USA). After 30 min incubation, the membranes were washed six times (5 min per wash) in TBST, then incubated with Supersignal Chemiluminescent Reagent (Pierce, Rockford, IL, USA) for 5 min and exposed to high-sensitivity chemiluminescence phosphor-imager screens (Bio-Rad) for 5 min, and the distribution and intensity of the fluorescence determined using a GS-250 Molecular Imager (Bio-Rad).

### RESULTS

The initial phase of our study determined whether the enhanced radioresistance of the 2780/CP cell line was attributable to a decrease in the level of DSBs induced by radiation and/or to an enhanced level of DSB rejoining. For these studies, we used a PFGE technique that did not require preincubation of the cells with radioisotopes, thereby eliminating any potential induction of radioresponsive processes or alteration in cell survival. The minor alterations in the cell cycle distributions of the two 2780 cell lines studied had no obvious impact on the level of DNA migration from the embedded nuclei. For unirradiated 2780/WT cells, 96.9 ± 0.7% ($n$ = 18) of the DNA was retained in the plug after electrophoresis, whereas for unirradiated 2780/CP cells 97.8 ± 0.9% ($n$ = 16) of the DNA was retained in the plug after electrophoresis. The low dose (i.e. < 10 Gy) resolution of the PFGE

**Table 2** Ampicillin-resistant bacterial clones generated after transfection of DH5α bacteria with *Eco*RI-digested pUC18 molecules after exposure to T4 ligase, or various nuclear protein extracts. (Values represent the mean values ± s.e.m. of data that have been corrected for 'background' plasmid rejoining by subtraction of internal control data)

| Treatment | Total colonies produced[a] | White colonies produced[a] | Total colonies produced[b] | Misrepair frequency |
|---|---|---|---|---|
| *Eco*RI only | 43.44 ± 13.22 | 0.055 ± 0.055 | 46.82 ± 21.07 | 0 |
| *Eco*RI→T4 ligase | 4950 ± 45.92 | 0.33 ± 0.31 | 5125 ± 67.23 | 0 |
| *Eco*RI → Nuclear protein → T4 ligase | 5744 ± 67.12 | 0.19 ± 0.08 | 5463 ± 79.19 | 0 |
| 2780/WT | 93.6 ± 15.09 | 14.3 ± 3.66 | 96.04 ± 15.50 | 15.06 ± 2.40 |
| 2780/CP | 126.7 ± 13.56 | 8.8 ± 1.39 | 121.80 ± 23.62 | 4.94 ± 0.95 |
| OAW42/WT | 113.7 ± 4.08 | 8.0 ± 0.53 | 116.00 ± 4.16 | 7.04 ± 0.35 |
| OAW42/CP | 141.4 ± 7.91 | 5.3 ± 0.74 | 133.40 ± 7.46 | 3.85 ± 0.54 |
| CHO-AA8 | 126.6 ± 21.60 | 4.3 ± 1.85 | 164.2 ± 27.77 | 3.18 ± 0.85 |

[a]Number of ampicillin-resistant bacterial colonies produced per $10^4$ bacterial cells plated. [b]Number of ampicillin-resistant bacterial colonies produced normalized to bacterial cell viability.

© Cancer Research Campaign 1999

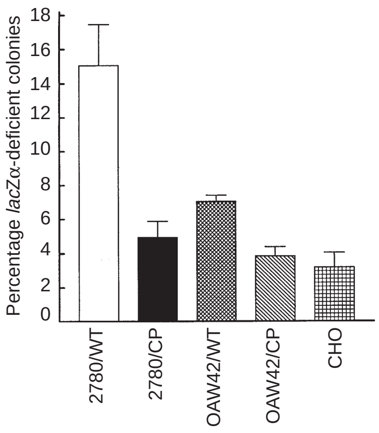

**Figure 3** DSB misrejoining activity in the human ovarian tumor cell lines and in CHO cells. Misrejoining capacity was assayed by at least three separate experiments protocol facilitated by phosphorimager analysis has allowed us to measure radiation-induced DSBs by PFGE after biologically relevant (i.e. survival curve) doses of radiation. The induction of DSBs was essentially linear with respect to dose in both the 2780/WT and 2780/CP cell lines (Figure 1). The level of DSB induction in the CHO-AA8 cell line was essentially identical to that observed when prelabelled CHO cells were used, suggesting that the sensitivity of the Alu+ based Southern blotting technique was comparable to our earlier PFGE protocol (Murray et al, 1994).

Having established that the induction of collateral radioresistance in the 2780/CP cell lines was not attributable to a reduction in the level of DSB induction (a possibility given that the 2780/CP line has a higher level of glutathione (GSH) than the parental 2780/WT cell line), we determined whether there were any differences in the capacity of these two cell lines to rejoin radiation-induced DSBs. As repair time increased, less DNA migration into the gel was observed in both cell lines; however, there were no significant differences in the level of residual DSBs present in the 2780/WT and 2780/CP cell lines over the 240-min time course (Figure 2). Finally, the reduction in radiosensitivity after the development of cisplatin resistance in the 2780/CP cell was not associated with any significant change in DNA-PKcs protein levels from those observed in the 2780/WT cell line (data not shown).

The level and fidelity of DSB rejoining effected by nuclear protein extracts from the 2780/WT and 2780/CP cell lines was then determined using our cell-free plasmid reactivation assay. There was no significant difference in the number of bacterial colonies produced after transformation of the DH5α cells with *Eco*RI-linearized pUC18 plasmid that had been exposed to nuclear protein extracts from the 2780/WT and 2780/CP cells (Table 2). However, the induction of cisplatin resistance in the 2780/CP cell line was associated with a threefold ($P < 0.01$) reduction in the frequency with which *lac*Zα-deficient (white) colonies were formed (Figure 3, Table 2). We established whether this reduction in DSB misrejoining activity after the induction of cisplatin resistance was a cell-line-specific phenomenon by establishing the fidelity of DSB rejoining in another human ovarian tumour cell line (OAW42/WT) and its cisplatin-resistant variant (OAW42/CP). *Eco*RI-linearized pUC18 plasmid that had been exposed to nuclear protein extracts from the OAW42/WT and OAW42/CP cells gave essentially identical levels of bacterial transformation (Table 2). Although there was approximately twofold less DSB misrejoining activity in the OAW42/WT than in the 2780/WT nuclear protein extracts, the induction of cisplatin resistance in the OAW42 cell line was also associated with a significant ($P < 0.001$) reduction in DSB misrejoining activity (Figure 3).

PCR analysis of six randomly selected white (mutant) colonies that were generated after exposure of the *Eco*RI-linearized pUC18 plasmid to 2780/WT nuclear protein revealed the inactivation of the *lac*Zα gene in all the clones studied was attributable to deletion events, giving a PCR product of between 184 and 494 bp. Sequencing of the 'mutant' pUC18 plasmids revealed that in five of the six clones the deletion arose via the illegitimate rejoining between distant short-sequence DNA repeats (microhomologies). The inactivation of the *lac*Zα gene in the other clone (W5) was attributable to a more complex deletion event: in this instance, nt 290–335 and nt 354–748 were deleted, the intervening 18-bp fragment (nt 336–353) was retained but had been inverted. The rejoining of the inverted 18-bp fragment appears to have occurred via illegitimate rejoining events between DNA microhomologies at either end of the fragment and the corresponding sequences at the termini of the pUC18 plasmid. Interestingly, approximately half of the randomly selected mutant clones had deletion break points close to nt 450 (the original *Eco*RI cleavage site) and to nt 700.

**Table 3** Characterization of the inactivating *lac*Zα mutations generated by 2780/WT nuclear protein extracts

| Bacterial clone | PCR product size (bp) | Nature of mutation | Deleted region (nt) | DNA sequence at deletion breakpoints |
|---|---|---|---|---|
| Blue | 628 | – | – | – |
| W1 | 494 | Deletion (134 bp) | 413–548 | GCCT |
| W2 | 380 | Deletion (248 bp) | 449–698 | CTC |
| W3 | 375 | Deletion (253 bp) | 450–703 | CTC |
| W4 | 377 | Deletion (251 bp) | 447–698 | GCT |
| W5 | 190 | Double deletion (43 bp, 395 bp), plus inversion insertion | 290–335, 354–748 | GC, AG |
| W6 | 184 | Deletion (444 bp) | 287–731 | CGGC |

© Cancer Research Campaign 1999 

### DISCUSSION

In this study, we have determined whether the cisplatin-induced collateral radioresistance that preferentially occurs in inherently radiosensitive human tumour cells was associated with a reduction or suppression of non-conservative DSB rejoining activity. Our data suggest that there is significantly ($P < 0.01$) lower DSB misrejoining activity within nuclear protein extracts from collaterally radioresistant (cisplatin-resistant) cell lines than in nuclear protein extracts from their respective cisplatin- and radiation-sensitive parental cell lines. This reduction in DSB misrejoining activity appears to be independent of the DSB rejoining capacity of the cell lines studied, and it is unlikely to be a manifestation of variations in cell cycle progression because these measurements were performed using a cell-free reactivation assay. Clearly, the relative distribution of the cells within the various cell cycle phases may have a major impact upon the composition of the nuclear protein extract, but there are no major differences in the cell cycle distributions of the cisplatin-sensitive and -resistant cell lines.

Acquired cisplatin resistance is frequently multifactorial, and it is likely that repeated exposure to cisplatin will induce a spectrum of genetic mutations that could conceivably alter the fidelity of DSB rejoining. For example, the induction of cisplatin resistance in both the 2780/CP and OAW42/CP cell lines was associated with a decrease in the functionality of the p53 protein (data not shown). Although circumstantially these data suggest that p53 functionality might be an important determinant of DSB misrejoining activity, recent studies have shown that the introduction of a mutant *p53* gene per se has no impact on DSB misrejoining activity (Bill et al, 1997). Another phenotypic difference that is consistently induced after the development of cisplatin resistance in both the 2780/CP and OAW42/CP cell lines is the reduction in MLH1 protein levels (data not shown). Differences in mismatch repair activity could potentially influence DSB misrejoining activity, because the mismatch repair pathway can repair DSBs provided there are mismatch repair tracts on both sides of the break (Weng and Nickoloff, 1997). However, our characterization of the nuclear protein extract-induced mutations within the *lac*Zα gene would suggest that the development of cisplatin resistance had altered processes other than mismatch repair capacity. Sequencing of the mutant 'white' plasmids generated in our experiments suggests that the inactivation of the *lac*Zα gene was attributable to deletions of between 136 and 444 bp, all of which involved combination at distant short sequence repeats (microhomologies). There is presently little information on the protein(s) responsible for the non-conservative DSB rejoining activity that our assay has detected. To date, only one protein complex has been purified from mammalian cells that is involved in non-homologous rejoining (Fishel et al, 1991). This protein complex is not fully characterized, but it is presently known to consist of the HPP-1, RPA (also known as human single-stranded DNA-binding protein-HSSB) and a unique ligase (but not I, II or III). It is possible that the plasmid reactivation assay has detected differences in the activity of this protein complex in the cisplatin-sensitive and -resistant cell lines.

The high level of illegitimate recombination effected by 2780/WT nuclear protein extracts raises the possibility that the sensitivity of the 2780/WT cells to cisplatin and radiation may be partly due to an abnormally high level of non-conservative rejoining activity. A proportion of cisplatin–DNA adducts do appear to be repaired through a non-homologous recombinational pathway (Cizeau et al, 1996). Some form of DNA recombination is certainly required to repair cisplatin interstrand cross-links, and it seems likely that non-conservative recombination would have a greater likelihood of leading to cell death (due to loss of critical DNA) than if the adducts were repaired via a homologous recombination activity. It is, therefore, conceivable that the development of cisplatin resistance arises through either the selection of cells that have a preferentially higher level of homologous than non-homologous recombination (NHR) activity, or the suppression of the NHR pathway. However, it would appear that the lower DSB misrejoining frequencies in the 2780/CP and OAW42/CP cell lines arose because of a genetic or epigenetic alteration induced during the development of cisplatin resistance because their respective parental cell lines were clonal in origin. Other than the data presented here, we are unaware of any data that directly suggests that radio- or cisplatin-sensitive human tumour cells have a higher level of NHR activity than their resistant counterparts.

In summary, the 2780 paired cell lines are another example of human tumour cell lines that differ greatly in radiosensitivity, yet show equal or similar levels of DSB rejoining (Schwartz and Vaughan, 1989; Smeets et al, 1993; McKay et al, 1995). These data in isolation would circumstantially support the notion that human tumour cell lines may differ in their tolerance to residual DNA damage, however, the DSB misrejoining data presented in this paper (Figure 3) and elsewhere (Schwartz and Vaughan, 1989; Powell and McMillan, 1991; Powell et al, 1992) suggest that the lethality of radiation-induced DSB may be determined by both the level and fidelity of DSB rejoining. These data support the notion that the radiosensitivity of DSB repair-proficient human tumour cell lines may be partly determined by the predisposition of these cell lines to activate non-conservative DSB rejoining pathways. Furthermore, our data suggest that the induction of acquired cisplatin resistance is associated with a two- to threefold decrease in the activity of a non-conservative DSB rejoining mechanism that appears to be a manifestation of NHR. These data provide a novel insight into the potential role that NHR activity might play as a predisposing factor to both radiation and cisplatin sensitivity in human tumour cells.

### ACKNOWLEDGEMENTS

This work was funded by grants to Dr Richard Britten from the Alberta Cancer Board-Research Initiative Program (RI-116). We are grateful to Dr David Murray for his advice and guidance with the Alu-based Southern Analysis of PFGE gels, and to Dr Joan Allalunis-Turner for the provision of the DNA-$PK_{cs}$ antibody.

### REFERENCES

Bill CA, Yu Y, Miselis NR, Little JB and Nickoloff JA (1997) A role for p53 in DNA end rejoining by human cell extracts. *Mutant Res* **385**: 21–29

Britten RA, Warenius HM, Masters JRW and Peacock JH (1993) The differential induction of collateral resistance to 62.5 MeV (p→$Be^+$) neutrons and 4 MeV photons by exposure to cis-platinum. *Int J Radiat Oncol Biol Phys* **26**: 837–843

Britten RA, Warenius HM, Carraway AV and Murray D (1994) Differential modulation of radiosensitivity following induction of *cis*-platinum resistance in radiation-sensitive and radiation-resistant human tumor cells. *Radiat Oncol Invest* **2**: 25–31

Britten RA, Liu D, Kuny S and Allalunis-Turner MJ (1997) Differential level of DSB repair fidelity effected by nuclear protein extracts derived from radiosensitive and radioresistant human tumour cells. *Br J Cancer* **76**: 1440–1447

© Cancer Research Campaign 1999

Bryant PE and Liu N (1994) Responses of radiosensitive repair-proficient cell lines to restriction endonucleases. *Int J Radiat Biol* **66**: 597–601

Cizeau J, Decoville M, Leng M and Locker D (1996) Large deletions in the *white* gene of *Drosophila melanogaster* by the antitumoral drug cis-dichlorodiammineplatinum (II): influence of non-homologous recombination. *Mutat Res* **356**: 197–202

Cox R, Masson WK, Debenham PG and Webb MBT (1984) The use of recombinant DNA plasmids for the determination of DNA-repair and recombination in cultured mammalian cells. *Br J Cancer* **49** (suppl. VI): 67–72

Fishel R, Derbyshire MK, Moore SP and Young CSH (1991) Biochemical studies of homologous and nonhomologous recombination in human cells. *Biochimie* **73**: 257–267

Ganesh A, North P and Thacker J (1993) Repair and misrepair of site-specific DNA double-strand breaks by human cell extracts. *Mutat Res* **299**: 251–259

Giaccia A, Weinstein R, Hu J and Stomato TD (1985) Cell cycle repair of double-strand DNA breaks in a gamma-ray-sensitive Chinese hamster cell. *Somat Cell Mol Genet* **11**: 485–491

Hida T, Ueda R, Takahashi T, Watanabe H, Kato T, Suyama M, Sugiura T, Ariyoshi Y and Takahashi T (1989) Chemosensitivity and radiosensitivity of small cell lung cancer cell lines studied by a newly developed 3-(4,5-dimethylthiazol-2-yl)-2,5-diphenyltetrazolium bromide (MTT) hybrid assay. *Cancer Res* **49**: 4785–4790

Kirchgessner CU, Patil CK, Evans JW, Cuomo CA, Fried LM, Carter T, Oettinger MA and Brown JM (1995) DNA-dependent kinase (p350) as a candidate gene for the murine SCID defect. *Science* **267**: 1178–1183

Lambin P, Coco-Martin J, Legal JD, Begg AC, Parmentier C, Joiner MC and Malaise EP (1994) Intrinsic radiosensitivity and chromosome aberration analysis using fluorescence in situ hybridization in cells of two human tumor cell lines. *Radiat Res* **138**: S40–43

Lees-Miller SP, Godbout R, Chan DW, Weinfeld M, Day RS, Barron GM and Allalunis-Turner J (1995) Absence of p350 subunit of DNA-activated protein kinase from a radiosensitive human cell line. *Science* **267**: 1183–1185

Liu N and Bryant PE (1993) Response of ataxia telangiectasia cells to restriction endonuclease induced DNA double-strand breaks. I. Cytogenetic characterization. *Mutagenesis* **8**: 503–510

Lou C-M, Tang W, Mekeel KL, DeFrank JS, Rani Anné P and Powell SN (1996) High frequency and error-prone DNA recombination in ataxia telangiectasia cell lines. *J Biol Chem* **271**: 4497–4503

Louie KG, Behrens BC, Kinsella TJ, Hamilton TC, Grotzinger KR, McKoy WM, Winker MA and Ozols RF (1985) Radiation survival parameters of antineoplastic drug-sensitive and -resistant human ovarian cell lines and their modification by buthionine sulfoximine. *Cancer Res* **45**: 2110–2115

McKay MJ and Kefford RF (1995) The spectrum of *in vitro* radiosensitivity in four human melanoma cell lines is not accounted for by differential induction or rejoining of DNA double strand breaks. *Int J Radiat Oncol Biol Phys* **31**: 345–352

Murray D, Simpson R, Rosenberg E, Carraway A and Britten R (1994) Correlation between γ-ray induced DNA double-strand breakage and cell killing after biologically relevant doses: analysis by pulsed-field gel electrophoresis. *Int J Radiat Biol* **65**: 419–426

Olive PL, Banáth JP and MacPhail HS (1994) Lack of a correlation between radiosensitivity and DNA double-strand break induction or rejoining in six human tumor cell lines. *Cancer Res* **54**: 3939–3946

Powell S and McMillan TJ (1991) Clonal variation of DNA repair in a human glioma cell line. *Radiother Oncol* **21**: 225–232

Powell SN and McMillan TJ (1994) The repair fidelity of restriction enzyme-induced double strand breaks in plasmid DNA correlates with radioresistance in human tumor cell lines. *Int J Radiat Oncol Biol Phys* **29**: 1035–1040

Powell SN, Whitaker SJ, Edwards SM and McMillan TJ (1992) A DNA repair defect in a radiation-sensitive clone of a human bladder carcinoma cell line. *Br J Cancer* **65**: 798–802

Powell SN, Whitaker S, Peacock JH and McMillan TJ (1993) Ataxia telangiectasia: an investigation of the repair defect in the cell line AT5BIVA by plasmid reconstitution. *Mutat Res* **294**: 9–20

Russell NS, Arlett CF, Bartelink H and Begg AC (1995) Use of fluorescence in situ hybridization to determine the relationship between chromosome aberrations and cell survival in eight human fibroblast strains. *Int J Radiat Biol* **68**: 185–196

Sasai K, Evans JW, Kovacs MS and Brown JM (1994) Prediction of human cell radiosensitivity: comparison of clonogenic assay with chromosome aberrations scored with premature chromosome condensation with fluorescence in situ hybridization. *Int J Radiat Oncol Biol Phys* **30**: 1127–1132

Schwartz JL and Vaughan ATM (1989) Association among DNA/chromosome break rejoining rates, chromatin structure alterations, and radiation sensitivity in human tumor cell lines. *Cancer Res* **49**: 5054–5057

Smeets MFMA, Mooren EHM and Begg AC (1993) Radiation-induced DNA damage and repair in radiosensitive and radioresistant human tumour cells measured by field inversion gel electrophoresis. *Int J Radiat Biol* **63**: 703–713

Taccioli GE, Gottlieb TM, Blunt T, Priestley A, Demengeot J, Mizuta R, Lehmann AR, Alt FW, Jackson SP and Jeggo PA (1994) Ku80: product of XRCC5 gene and its role in DNA repair and V(D)J recombination. *Science* **265**: 1442–1445

Thacker J and Ganesh AN (1990) DNA-break repair, radioresistance of DNA synthesis, and camptothecin sensitivity in the radiation-sensitive *irs* mutants: comparisons to ataxia-telangiectasia cells. *Mutat Res* **235**: 49–58

Thacker J, Chalk J, Ganesh A and North P (1992) A mechanism for deletion formation in DNA by human cell extracts: the involvement of short sequence repeats. *Nucleic Acids Res* **20**: 6183–6188

Turchi JJ, Patrick SM and Henkels KM (1997) Mechanisms of DNA-dependent protein kinase inhibition by *cis*-diamminedichloroplatinum(II)-damaged DNA. *Biochemistry* **36**: 7586–7593

Twentyman PR, Wright KA and Rhodes T (1991) Radiation response of human lung cancer cells with inherent and acquired resistance to cisplatin. *Int J Radiat Oncol Biol Phys* **20**: 217–220

Weng G and Nickoloff J (1997) Conversion on opposite sides of a double-strand break can be mediated by two mismatch repair tracts. *Proc Am Assoc Cancer Res* **38**: A2399

*© Cancer Research Campaign 1999*
